# Natural History of Psychological Symptoms in Individuals With Rome IV Irritable Bowel Syndrome and Association With Gastrointestinal Symptom Severity

**DOI:** 10.1111/nmo.70301

**Published:** 2026-03-30

**Authors:** Mais Khasawneh, Vivek C. Goodoory, Alexander C. Ford, Christopher J. Black

**Affiliations:** ^1^ Leeds Institute of Medical Research at St. James's University of Leeds Leeds UK; ^2^ Leeds Gastroenterology Institute St. James's University Hospital Leeds UK

**Keywords:** irritable bowel syndrome, latent class analysis, mood, somatization, subgrouping

## Abstract

**Background and Aims:**

Psychological symptoms are common in irritable bowel syndrome (IBS) and are associated with more severe gastrointestinal symptoms. However, data describing the relationship between psychological symptoms and IBS symptom severity, and how this changes over time, are limited. We aimed to characterize the longitudinal course of psychological symptoms in individuals with Rome IV IBS and examine their association with gastrointestinal symptom severity over the same time period.

**Methods:**

We recruited individuals with self‐reported IBS from three UK community‐based organizations who completed validated questionnaires assessing gastrointestinal symptoms and psychological symptoms at baseline and after 12 months. IBS symptom severity was measured using the IBS Severity Scoring System, and psychological symptoms were assessed using the Hospital Anxiety and Depression Scale, Patient Health Questionnaire‐12, Visceral Sensitivity Index, and Perceived Stress Scale. Analyses included participants meeting Rome IV criteria at both time points.

**Results:**

Among 1375 individuals recruited, 811 (59.0%) met Rome IV criteria at baseline. Of these 811 individuals who met Rome IV criteria at baseline, 452 (55.7%) provided follow‐up data at 12 months and were the population of interest for this study. All psychological symptoms demonstrated moderate stability over 12 months (κ = 0.43–0.50, % agreement 61.7%–69.5%). Higher levels of anxiety, depression, extraintestinal symptom reporting, gastrointestinal symptom‐specific anxiety, and perceived stress were consistently observed among individuals with persistently moderate‐to‐severe IBS symptoms, while those with worsening IBS symptoms also showed increased psychological symptom burden over time.

**Conclusions:**

Psychological symptoms are moderately stable and closely associated with gastrointestinal symptom severity in Rome IV IBS. These findings support integrated gut–brain behavioral approaches to treatment and longitudinal assessment of psychological symptoms as part of the routine clinical management of IBS.

## Key Points

- Psychological symptoms in individuals with Rome IV irritable bowel syndrome show moderate stability over 12 months.
- Greater psychological symptom burden is associated with increased IBS symptom severity.
- Psychological symptom trajectories mirror changes in gastrointestinal symptom severity over time, consistent with gut–brain interaction in IBS.

## Graphical abstract

Psychological symptoms in Rome IV irritable bowel syndrome closely track gastrointestinal symptom severity. Worsening IBS is accompanied by increasing psychological burden, supporting integrated gut–brain approaches to longitudinal clinical management.

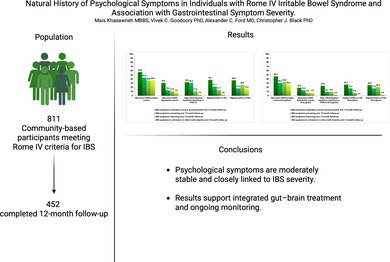

**Abbreviations**
IBS: Irritable bowel syndrome
HADS: Hospital Anxiety and Depression Scale
IBS‐SSS: IBS Severity Scoring System
PHQ: Patient Health Questionnaire
PSS: Perceived Stress Scale
VSI: Visceral Sensitivity Index

## 1 Introduction

Irritable bowel syndrome (IBS) is a common disorder of gut–brain interaction characterized by abdominal pain and alteration in stool frequency or form [1]. It is associated with substantial symptom burden, impaired quality of life, and increased health care utilization [2, 3]. The Rome IV criteria identify a more severe phenotype of patients than earlier iterations of the Rome criteria, characterized by greater gastrointestinal symptom severity and higher rates of psychological comorbidity [4, 5]. Psychological symptoms, including anxiety, depression, and somatoform symptom‐reporting, are highly prevalent in IBS and are increasingly recognized as core features of the disorder for some individuals, rather than secondary consequences of chronic gastrointestinal symptoms [6, 7, 8].

Published literature demonstrates strong and consistent associations between psychological symptom burden and IBS severity, disease impact, and quality of life, with gastrointestinal symptom‐specific anxiety and somatoform symptom‐reporting emerging as particularly relevant constructs [9, 10]. Longitudinal follow‐up studies of individuals with Rome IV IBS further suggest that psychological comorbidity is both relatively stable over time and identifies subgroups with more severe and persistent symptoms [11, 12, 13]. However, most of these follow‐up studies have focused on the presence of psychological comorbidity at baseline as a predictor of prognosis. There has been little work examining how psychological symptoms and gastrointestinal symptom severity change together over time.

Understanding the longitudinal relationship between psychological symptom trajectories and gastrointestinal symptom severity in IBS is clinically important, as it may inform risk stratification and support the use of integrated gut–brain management strategies in a subset of patients. Therefore, the aim of this study was to characterize the longitudinal course of psychological symptoms in individuals with Rome IV‐defined IBS and to examine how these associate with changes in gastrointestinal symptom severity over time. We hypothesized that psychological symptom burden would be relatively stable over time and be independently associated with gastrointestinal symptom severity.

## 2 Methods

### 2.1 Participants and Setting

We recruited participants who self‐identified as having IBS from three UK organizations. These were the IBS network, the registered charity for individuals living with the condition, TalkHealth, an online social health community providing information about various medical conditions, and ContactMe‐IBS, a research registry for individuals with IBS who are not presently under specialist care. Data from this cohort have been published previously [8, 10, 13, 14]. We invited individuals registered with these organizations to participate in the initial study via e‐mail and post, between December 2017 and December 2018. The correspondence directed potential participants to a study website providing detailed information about the research. Individuals wishing to take part completed a web‐based questionnaire, with responses stored in a secure online database. A reminder was sent to nonresponders to complete the questionnaire. The questionnaire collected demographic data and assessed lower gastrointestinal symptoms, extraintestinal symptoms, and psychological health. After a minimum interval of 12 months, all participants were recontacted via the same communication channels and invited to complete a follow‐up questionnaire. This was identical to the baseline questionnaire. There was no reward or incentive to complete either questionnaire. There were no exclusion criteria, other than an inability to understand written English. The University of Leeds research ethics committee approved the study in November 2017 (MREC17–018).

### 2.2 Data Collection and Synthesis

#### 2.2.1 Demographic and Symptom Data

We collected demographic information, including age, sex, ethnicity, marital status, and educational attainment, and asked participants to indicate whether they had consulted a primary care physician or a gastroenterologist regarding their IBS symptoms. We assessed lower gastrointestinal symptoms using the Rome IV questionnaire, [15] and classified each participant as meeting or not meeting Rome IV criteria for IBS according to the recommended scoring algorithm. Symptom severity was evaluated using the IBS Severity Scoring System (IBS‐SSS), [16] which assesses the presence, frequency, and severity of abdominal pain, the presence and severity of abdominal distension, satisfaction with bowel habits, and the extent to which IBS symptoms interfere with daily life. Scores range from 0 to 500, with values < 75 indicating symptom remission, 75–174 indicating mild symptoms, 175–299 moderate symptoms, and 300–500 severe symptoms.

#### 2.2.2 Assessment of Psychological Symptoms

We assessed symptoms of anxiety and depression using the Hospital Anxiety and Depression Scale (HADS) [17]. The scale consists of 14 items, with seven each for anxiety and depression. Items are scored from 0 to 3, producing subscale scores between 0 and 21. Severity is categorized as normal (0–7), borderline abnormal (8–10), or abnormal (≥ 11) for both the anxiety and depression subscales.

We assessed extraintestinal symptoms using the Patient Health Questionnaire‐12 (PHQ‐12), [18] which is derived from the validated PHQ‐15 [19]. The total PHQ‐12 score ranges from 0 to 24. We categorized severity into high (total PHQ‐12 ≥ 13), medium (8–12), low (4–7), or minimal (≤ 3) levels.

We measured gastrointestinal symptom‐specific anxiety using the 15‐item Visceral Sensitivity Index (VSI) [9]. Each item is rated on a six‐point scale ranging from strongly disagree (Scores 0) to strongly agree (Scores 5). In view of the absence of a validated cutoff score to categorize levels of gastrointestinal symptom‐specific anxiety, we divided these data into tertiles of equal size.

We assessed perceived stress using the 10‐item Perceived Stress Scale (PSS), [20] derived from the original 14‐item version [21]. The PSS is considered psychometrically reliable and comparable to the full scale and evaluates the extent to which individuals have felt stressed during the preceding 1 month. As with the VSI, no validated cutoff exists for defining low, medium, or high perceived stress. Therefore, we also divided PSS scores into equal‐sized tertiles.

### 2.3 Statistical Analysis

We included only participants who met the Rome IV criteria for IBS at both baseline and 12‐month follow‐up. We evaluated the stability of psychological and somatic symptom classifications over time, by comparing baseline and follow‐up categories for HADS‐anxiety, HADS‐depression, PHQ‐12, VSI, and PSS. The degree of agreement between baseline and follow‐up for each marker of psychological health was assessed using Cohen's kappa statistic. Values of 0 to 0.20 indicate slight agreement, 0.21 to 0.40 fair agreement, 0.41 to 0.60 moderate agreement, 0.61 to 0.80 substantial agreement, and 0.81 to 1 almost perfect agreement. We also calculated the % agreement between baseline and follow‐up for each marker of psychological health. We then compared the proportions with abnormal HADS‐anxiety or HADS‐depression scores, high PHQ‐12 scores, and in the highest tertile for VSI or PSS scores at 12‐month follow‐up according to IBS symptom severity trajectory groups over the 12 months of follow‐up (remission or mild IBS symptoms at both baseline and 12‐month follow‐up, improving IBS symptoms over 12‐month follow‐up (moderate‐to‐severe symptoms at baseline with remission or mild symptoms at 12‐month follow‐up), worsening IBS symptoms over 12‐month follow‐up (remission or mild symptoms at baseline with moderate‐to‐severe symptoms at 12‐month follow‐up), and moderate‐to‐severe IBS symptoms at both baseline and 12‐month follow‐up). Finally, we compared the proportions with abnormal HADS‐anxiety or HADS‐depression scores, high PHQ‐12 scores, and in the highest tertile for VSI or PSS scores at both baseline and 12‐month follow‐up according to IBS symptom severity trajectory groups over the 12 months of follow‐up. We examined these associations using a Pearson χ^2^ test. Due to multiple comparisons, a *p* value of < 0.01 was used to define statistical significance. We used SPSS version 30.0 (SPSS Inc., Chicago, IL, USA) for all analyses.

## 3 Results

We recruited 1375 individuals who self‐identified as having IBS from the three organizations. Of these 1375 individuals, 811 (59.0%) met Rome IV criteria for IBS at baseline. In total, 784 (57.0%) of 1375 individuals were followed up successfully at 12 months and provided complete symptom data, and 591 were not. Demographic data of those who did and did not respond to the 12‐month questionnaire have been reported elsewhere [13] and are provided in Table S1. Among the 811 participants who met Rome IV criteria for IBS at baseline, 452 (55.7%) were followed up successfully at 12 months and provided complete symptom data (mean age 49.1 years; 386 [85.4%] female). These 452 individuals served as the population of interest for this study, of whom 319 (70.6%) still met the Rome IV criteria for IBS at 12 months.

### 3.1 Natural History of Anxiety Symptoms

**FIGURE 1:**
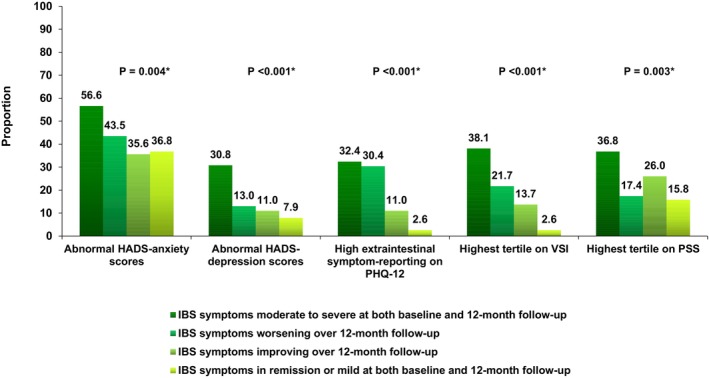
Proportion of individuals with Rome IV IBS with abnormal HADS‐anxiety scores, abnormal HADS‐depression scores, highest extraintestinal symptom‐reporting on the PHQ‐12, highest tertile on the VSI, and highest tertile on the PSS at 12‐month follow‐up according to IBS symptom severity trajectories. Proportion: *p* < 0.001*, *p* = 0.004*, *p* < 0.001*, *p* < 0.001*, *p* = 0.003*. *p value for Pearson χ^2^.

**FIGURE 2:**
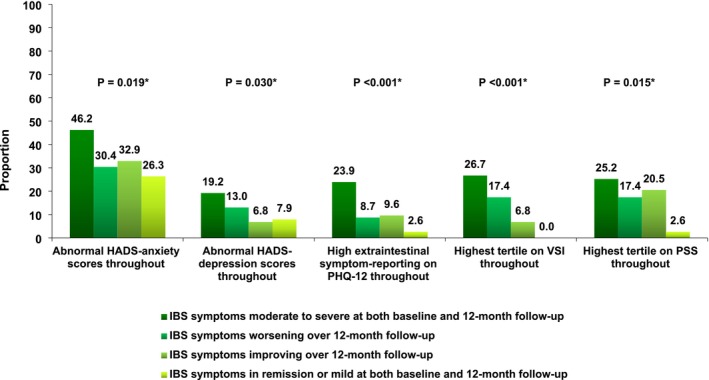
Proportion of individuals with Rome IV IBS with abnormal HADS‐anxiety scores, abnormal HADS‐depression scores, highest extraintestinal symptom‐reporting on the PHQ‐12, highest tertile on the VSI, or highest tertile on the PSS at both baseline and 12‐month follow‐up according to IBS symptom severity trajectories. Proportion: p = 0.030*, *p* = 0.019*, *p* < 0.001*, *p* < 0.001*, *p* = 0.015*. *p value for Pearson χ^2^.

There was moderate agreement in HADS‐anxiety scores between baseline and 12‐month follow‐up (Kappa = 0.45, % agreement = 66.4%). Among 115 individuals with normal scores at baseline 85 (73.9%) continued to exhibit normal scores at 12 months and among 246 with abnormal scores 188 (76.4%) continued to report abnormal scores at 12 months (*p* < 0.001 for trend). HADS‐anxiety scores at 12‐month follow‐up varied significantly across IBS symptom severity trajectories. In total, 14 (36.8%) of 38 with remission or mild IBS symptoms at both baseline and 12‐month follow‐up reported abnormal HADS‐anxiety scores, 26 (35.6%) of 73 with improving symptoms over 12‐month follow‐up, 10 (43.5%) of 23 with worsening IBS symptoms over 12‐month follow‐up, and 180 (56.6%) of 318 with moderate‐to‐severe symptoms at both baseline and 12‐month follow‐up (*p* = 0.004 for trend) (Figure 1). Abnormal HADS‐anxiety scores at both baseline and 12 months were more likely in those with moderate to severe IBS symptoms at both baseline and 12‐month follow‐up (147 (46.2%) of 318 individuals), although this did not reach statistical significance (*p* = 0.019 for trend) (Figure 2).

### 3.2 Natural History of Depression Symptoms

Again, there was moderate agreement in HADS‐depression scores between baseline and 12‐month follow‐up (Kappa = 0.50, % agreement = 69.5%). Among 248 individuals with normal baseline scores, 194 (78.2%) remained normal at follow‐up, and among 97 with abnormal depression scores at baseline, 72 (74.2%) continued to report abnormal scores at 12 months (*p* < 0.001 for trend). HADS‐depression scores at follow‐up differed significantly across IBS symptom severity trajectories. Abnormal HADS‐depression scores were reported by three (7.9%) of 38 individuals with remission or mild IBS symptoms at both baseline and 12‐month follow‐up, eight (11.0%) of 73 with improving symptoms over 12‐month follow‐up, three (13.0%) of 23 with worsening symptoms over 12‐month follow‐up, and 98 (30.8%) of 318 with moderate‐to‐severe symptoms at both baseline and 12‐month follow‐up (*p* < 0.001 for trend) (Figure 1). Abnormal HADS‐depression scores at both baseline and 12 months were more likely in those with moderate to severe IBS symptoms at both baseline and 12‐month follow‐up (61 (19.2%) of 318 individuals), although again this did not reach statistical significance (*p* = 0.030 for trend) (Figure 2).

### 3.3 Natural History of Extra‐Intestinal Symptoms

Extraintestinal symptom‐reporting categories showed moderate agreement between baseline and follow‐up (Kappa = 0.44, % agreement = 61.9%). Of the 22 individuals with low baseline extraintestinal symptom‐reporting, 13 (59.1%) remained low at follow‐up; among 102 with mild levels, 66 (64.7%) remained mild. Among 199 with medium levels, 115 (57.8%) remained medium, and among 129 with high, 86 (66.7%) continued to exhibit high levels at 12 months (*p* < 0.001 for trend). Extraintestinal symptom‐reporting showed significant variability across IBS symptom severity trajectories. High levels were reported in one (2.6%) of 38 with remission or mild IBS symptoms at both baseline and 12‐month follow‐up, eight (11.0%) of 73 with improving symptoms over 12‐month follow‐up, seven (30.4%) of 23 with worsening symptoms over 12‐month follow‐up, and 103 (32.4%) of 318 with moderate‐to‐severe symptoms at both baseline and 12‐month follow‐up (*p* < 0.001 for trend) (Figure 1). High levels of extra‐intestinal symptom‐reporting at both baseline and 12‐month follow‐up were more likely in those with moderate‐to‐severe IBS symptoms at both baseline and 12‐month follow‐up (76 (23.9%) of 318 individuals, *p* < 0.001 for trend) (Figure 2).

### 3.4 Natural History of Gastrointestinal Symptom‐Specific Anxiety Symptoms

Gastrointestinal symptom‐specific anxiety also demonstrated moderate agreement from baseline to 12‐month follow‐up (Kappa = 0.43, % agreement = 62.2%). Of 156 individuals in the lowest tertile at baseline, 121 (77.6%) remained in the lowest tertile at follow‐up, and among 138 in the highest tertile, 94 (68.1%) remained in the highest tertile at 12 months (*p* < 0.001 for trend). The highest levels of gastrointestinal symptom‐specific anxiety were observed in one (2.6%) of 38 individuals with remission or mild IBS symptoms at both baseline and 12‐month follow‐up, 10 (13.7%) of 73 with improving symptoms over 12‐month follow‐up, five (21.7%) of 23 with worsening symptoms over 12‐month follow‐up, and 121 (38.1%) of 318 with moderate‐to‐severe symptoms at both baseline and 12‐month follow‐up (*p* < 0.001 for trend) (Figure 1). High levels of gastrointestinal symptom‐specific anxiety at both baseline and 12 months were more likely in those with moderate‐to‐severe IBS symptoms at both baseline and 12‐month follow‐up (85 (26.7%) of 318 individuals, *p* < 0.001 for trend) (Figure 2).

### 3.5 Natural History of Perceived Stress Symptoms

Finally, perceived stress symptoms demonstrated moderate agreement over 12 months (Kappa = 0.43, % agreement = 61.7%). Among 157 individuals in the lowest tertile at baseline, 108 (68.8%) remained in the lowest tertile at follow‐up, and among 145 in the highest tertile, 100 (69.0%) remained in the highest tertile at 12 months (*p* < 0.001 for trend). Perceived stress at 12 months differed across IBS symptom–severity trajectories. The highest levels of perceived stress symptoms were observed in six (15.8%) of 38 individuals with remission or mild IBS symptoms at both baseline and 12‐month follow‐up, 19 (26.0%) of 73 with improving symptoms over 12‐month follow‐up, four (17.4%) of 23 with worsening symptoms over 12‐month follow‐up, and 117 (36.8%) of 318 with moderate‐to‐severe symptoms at both baseline and 12‐month follow‐up (*p* = 0.003 for trend) (Figure 1). High levels of perceived stress symptoms at both baseline and 12 months were more likely in those with moderate‐to‐severe IBS symptoms at both baseline and 12‐month follow‐up (80 (25.2%) of 318 individuals), but this was not statistically significant (*p* = 0.015 for trend) (Figure 2).

## 4 Discussion

This longitudinal follow‐up study examined psychological symptoms and their natural history in individuals with Rome IV IBS, as well as their association with IBS symptom severity over 12 months. All psychological symptoms studied were moderately stable. Between 60% and 80% with symptoms at the lowest level remained in that category at 12‐month follow‐up. Between two‐thirds and three‐quarters of those with symptoms at the highest levels remained in that category at 12 months. The highest levels of psychological symptoms across all the questionnaires studied were seen in those with moderate‐to‐severe IBS symptoms at both baseline and 12‐month follow‐up and, with the exception of HADS‐anxiety scores, the lowest levels of psychological symptoms were seen in those individuals with remission or mild IBS symptoms at both baseline and 12‐month follow‐up. Individuals whose symptoms worsened over time also demonstrated a greater prevalence of psychological symptoms that were at the highest level, although to a lesser degree than those with persistently severe symptoms. Finally, a strong association was seen between the presence of psychological symptoms at the highest level at both baseline and 12‐month follow‐up the 12 months of the study and moderate‐to‐severe IBS symptoms at both baseline and 12‐month follow‐up, supporting their relevance to the chronicity of IBS symptoms.

The study recruited a large community‐based sample of individuals who self‐identified as having IBS. Most participants had consulted a primary care physician, a smaller proportion had seen a gastroenterologist, and only a minority had not sought medical advice for their symptoms. This broad recruitment approach increases confidence that the study population is applicable to a wide range of individuals living with IBS. In addition, the use of a web‐based questionnaire facilitated complete data collection for most variables of interest at both baseline and 12‐month follow‐up. Finally, we ensured that all individuals contributing data to this analysis met the Rome IV criteria for IBS at both time points.

Limitations should be acknowledged. The web‐based nature of the survey means that individuals with higher levels of internet access were likely recruited, which may have implications for the cohort under study, in terms of gastrointestinal and psychological health. We were unable to confirm the diagnosis of IBS in all participants using medical records; instead, this was based on self‐report and fulfillment of currently accepted diagnostic criteria. Although organic gastrointestinal diseases such as coeliac disease or inflammatory bowel disease that IBS can mimic, their prevalence in the community is substantially lower. Moreover, more than 95% of participants had consulted a physician regarding their symptoms, suggesting that most would have undergone some degree of clinical evaluation to exclude organic gastrointestinal pathology and were, therefore, likely to have IBS. We were unable to assess the influence of other medically unexplained comorbidities, such as fibromyalgia or chronic fatigue, or medications like gut–brain neuromodulators for these, and other, conditions, which may impact on both gastrointestinal and psychological symptoms. Responders at 12 months differed modestly from nonresponders, being older, more likely to be married or cohabiting or to have reached university or postgraduate education, less likely to smoke, more likely to identify as White Caucasian, and more likely to have consulted a doctor for their symptoms. Although these differences may limit the representativeness of the follow‐up sample, comparisons of responders with the original cohort revealed no significant differences in IBS symptom profiles, IBS symptom severity, or psychological symptom scores, other than for HAS‐depression scores, which were significantly higher among those not followed up successfully. In addition, the absolute demographic differences observed were relatively small, supporting the validity of longitudinal comparisons, and follow‐up rates were similar to other longitudinal studies of this type [22, 23, 24, 25, 26]. We were only able to assess the natural history of these symptoms at two points in time. Their longer‐term course is, therefore, uncertain. Finally, although diagnostic cutoffs for HADS and PHQ scores are widely accepted, this is not the case for VSI and PSS scores. Our decision to divide these into tertiles could, therefore, be criticized as information is lost.

Longitudinal studies in Rome IV‐defined IBS are consistent in demonstrating a substantial degree of psychological comorbidity in some individuals, with anxiety, depression, and somatoform symptom‐reporting showing relative stability over time and strong associations with gastrointestinal symptom severity and disease impact [11, 27]. Prior work has further identified gastrointestinal symptom‐specific anxiety and somatoform symptom‐reporting as particularly strong correlates of IBS severity and impaired quality of life, underscoring their central role in gut–brain interaction models of IBS [10]. In addition, a novel cluster model of IBS suggests that individuals with higher levels of psychological comorbidity constitute a distinct, and relatively stable, subgroup characterized by more severe and persistent gastrointestinal symptoms [8]. However, the majority of these studies focus on psychological symptoms at baseline as predictors of prognosis or diagnostic stability, providing limited insight into the longitudinal association between psychological symptom trajectories and gastrointestinal symptom severity trajectories. The present study extends this literature by providing some of the strongest longitudinal evidence, to date, that for many individuals degree of psychological symptom‐reporting and IBS symptom severity mirror each other over time. Therefore, our findings suggest that psychological distress functions as a dynamic component of the IBS disease process, rather than solely as a static vulnerability marker. Nevertheless, this is not the case for all individuals with IBS, as evidenced by the results of this study, where a substantial minority of patients had low levels of psychological comorbidity at both time points.

In conclusion, this study provides robust longitudinal evidence of a pivotal association between psychological symptom burden and IBS symptom severity in individuals with Rome IV‐defined IBS, reinforcing the need for integrated gut–brain care in routine clinical practice. These findings support systematic screening and ongoing monitoring of psychological symptoms in people with IBS to better inform risk stratification, treatment planning, and longitudinal gastrointestinal symptom management.

## Author Contributions

M.K., V.C.G., A.C.F., and C.J.B. conceived and drafted the study. C.J.B. and V.C.G. collected all data. M.K., C.J.B., and A.C.F. analyzed and interpreted the data. M.K. drafted the manuscript. All authors have approved the final draft of the manuscript.

## Funding

The authors have nothing to report.

## Conflicts of Interest

The authors declare no conflicts of interest.

## Supporting information


**Table S1:** Baseline characteristics of individuals responding to the 12‐month questionnaire compared with nonresponders.

## Data Availability

The data that support the findings of this study are available from the corresponding author upon reasonable request.
