# Supplementary material for: Natural History of Psychological Symptoms in Individuals With Rome IV Irritable Bowel Syndrome and Association With Gastrointestinal Symptom Severity
Source: Neurogastroenterol Motil. 2026 Mar 30;38(4):e70301. doi: 10.1111/nmo.70301 (PMC13035918; doi:10.1111/nmo.70301)
Supplement: Supplementary file 1 — Table S1: Baseline characteristics of individuals responding to the 12‐month questionnaire compared with nonresponders. [file NMO-38-e70301-s001.docx]

**Supplementary Table 1. Baseline Characteristics of Individuals Responding to the 12-month Questionnaire Compared with Non-responders.**

|  | **Responded to Questionnaire at 12 months**  **(n=784)** | **Did not Respond to Questionnaire at 12 months**  **(n = 591)** | **P value*** |
| --- | --- | --- | --- |
| **Mean age at study entry (SD)** | 50.7 (14.4) | 47.1 (16.4) | <0.001 |
| **Female gender (%)** | 660 (84.2) | 497 (84.1) | 0.96 |
| **Married or co-habiting at study entry (%)** | 535 (68.2) | 363 (61.4) | 0.009 |
| **University or postgraduate level of education (%)** | 369 (47.1) | 218 (37.2) | <0.001 |
| **Smoker at study entry (%)** | 49 (6.3) | 71 (12.0) | <0.001 |
| **White Caucasian ethnicity (%)** | 754 (96.2) | 539 (91.7) | <0.001 |
| **IBS after acute enteric infection (%)** | 102 (13.0) | 78 (13.2) | 0.90 |
| **Previously seen a GP regarding IBS at study entry (%)** | 754 (96.2) | 548 (92.9) | 0.007 |
| **Previously seen a gastroenterologist regarding IBS at study entry (%)** | 475 (60.6) | 314 (53.2) | 0.006 |
| **Rome IV criteria for IBS met at study entry (%)** | 452 (57.7) | 359 (60.8) | 0.24 |
| **IBS subtype at study entry (%)**  Constipation  Diarrhoea  Mixed stool pattern  Unclassified | 146 (18.6)  310 (39.5)  296 (37.8)  32 (4.1) | 124 (21.0)  207 (35.1)  220 (37.3)  35 (5.9) | 0.03 |
| **Severity on IBS-SSS at study entry (%)**  Remission  Mild  Moderate  Severe | 27 (3.4)  183 (23.3)  314 (40.1)  260 (33.2) | 28 (4.8)  110 (18.7)  231 (39.2)  220 (37.4) | 0.09 |
| **Mean HADS-anxiety score at study entry (SD)** | 10.0 (4.6) | 10.5 (4.8) | 0.05 |
| **Mean HADS-depression score at study entry (SD)** | 6.7 (4.4) | 7.4 (4.5) | 0.005 |
| **Mean PHQ-12 score at study entry (SD)** | 9.2 (4.0) | 9.7 (4.7) | 0.05 |
| **Mean VSI score at study entry (SD)** | 45.4 (17.8) | 47.1 (18.7) | 0.09 |
| **Mean PSS score at study entry (SD)** | 19.9 (8.2) | 20.9 (8.5) | 0.03 |

*P value for independent samples *t*-test for continuous data and Pearson χ^2^ for comparison of categorical data.
